# Non-invasive cardiovascular magnetic resonance pulmonary capillary wedge pressure predicts future atrial fibrillation: a LOOP substudy

**DOI:** 10.1093/eschf/xvaf038

**Published:** 2026-01-19

**Authors:** Maria Kalaitzoglou, Litten Bertelsen, Bradley Chambers, Gareth Matthews, Panagiotis Tsiverdis, Raluca Tomoaia, Thomas Anderton, Søren Zöga Diederichsen, Jesper Hastrup Svendsen, Pankaj Garg, Peter P Swoboda

**Affiliations:** Leeds Institute of Cardiovascular and Metabolic Medicine, University of Leeds, Leeds, UK; Department of Cardiology, Copenhagen University Hospital—Rigshospitalet, Copenhagen, Denmark; Leeds Institute of Cardiovascular and Metabolic Medicine, University of Leeds, Leeds, UK; Norwich Medical School, University of East Anglia, Norwich Research Park, Rosalind Franklin Road, Norwich Research Park, Norwich NR4 7UQ, UK; Department of Cardiology, Norfolk and Norwich University NHS Foundation Trust, Colney Lane, Norwich, NR4 7UY, Norfolk, UK; Leeds Institute of Cardiovascular and Metabolic Medicine, University of Leeds, Leeds, UK; Leeds Institute of Cardiovascular and Metabolic Medicine, University of Leeds, Leeds, UK; Leeds Institute of Cardiovascular and Metabolic Medicine, University of Leeds, Leeds, UK; Department of Cardiology, Copenhagen University Hospital—Rigshospitalet, Copenhagen, Denmark; Department of Cardiology, Copenhagen University Hospital—Rigshospitalet, Copenhagen, Denmark; Department of Clinical Medicine, Faculty of Health and Medical Sciences, University of Copenhagen, Copenhagen, Denmark; Norwich Medical School, University of East Anglia, Norwich Research Park, Rosalind Franklin Road, Norwich Research Park, Norwich NR4 7UQ, UK; Department of Cardiology, Norfolk and Norwich University NHS Foundation Trust, Colney Lane, Norwich, NR4 7UY, Norfolk, UK; Leeds Institute of Cardiovascular and Metabolic Medicine, University of Leeds, Leeds, UK

**Keywords:** Humans, Atrial fibrillation, Pulmonary capillary wedge pressure, Magnetic resonance imaging, Risk assessment, Follow-up studies

## Abstract

**Introduction:**

Elevated pulmonary capillary wedge pressure (PCWP) is known to drive atrial fibrillation (AF). However, it remains unknown if non-invasive cardiovascular magnetic resonance (CMR)-derived PCWP could predict the future risk of AF. This study investigated whether a CMR-derived measure of PCWP could predict future AF.

**Methods:**

We enrolled 202 participants (mean age 76.2 ± 4.2 years) from the LOOP study, each receiving implantable loop recorder for continuous rhythm monitoring over 4 years. Cardiovascular magnetic resonance imaging quantified left atrial volume (LAV) and left ventricular mass, allowing calculation of a validated sex-specific equation derived PCWP. Cox proportional hazards analysis identified independent variables associated with incident AF.

**Results:**

Eighty-six participants (42.6%) manifested AF during follow-up. Individuals with AF exhibited significantly higher CMR-PCWP (16.1 ± 2.8 vs 14.7 ± 2.3 mmHg, *P* < .01) and greater LAV. Univariate regression highlighted that PCWP ≥16 mmHg was significantly associated with incident AF [hazard ratio (HR): 2.73]. Stepwise Cox regression confirmed that PCWP ≥16 mmHg and the CHARGE-AF score remained independently associated with AF, with PCWP conveying higher HR (2.88, *P* < .001). Kaplan–Meier analysis reinforced the importance of this threshold for AF onset, demonstrating a significantly increased probability of arrhythmia over time and emphasizing its decisive clinical impact.

**Conclusion:**

Elevated CMR-PCWP is associated with AF in older, high-stroke-risk individuals, underscoring the role of subclinical diastolic dysfunction in promoting arrhythmogenesis. Incorporating non-invasive PCWP assessment into routine CMR evaluation may enhance risk stratification, allowing prompt identification of at-risk patients and enabling earlier, precise, targeted measures for AF prevention.

## Graphical Abstract

**Graphical Abstract:**
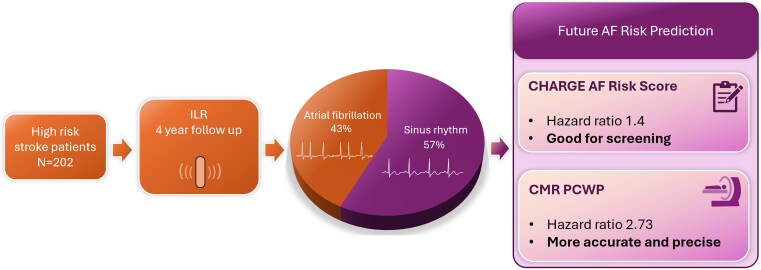
Long-term monitoring of high-risk stroke patients revealed a 43% incidence of atrial fibrillation, with cardiovascular magnetic resonance-derived pulmonary capillary wedge pressure providing superior risk prediction over CHARGE-AF risk score.

## Introduction

Atrial fibrillation (AF) is the most common sustained cardiac arrhythmia, with its prevalence rising sharply in individuals over the age of 70. Given its strong association with stroke, heart failure (HF), and mortality,^1^ accurate prediction of AF in high-risk individuals is critical for early intervention and risk mitigation. Current risk stratification models, such as the CHARGE-AF risk score,^2^ incorporate clinical and demographic variables but lack direct haemodynamic markers, potentially limiting their predictive accuracy.

Cardiovascular magnetic resonance (CMR)-derived pulmonary capillary wedge pressure (PCWP) has emerged as a non-invasive metric for assessing left ventricular (LV) filling pressure (FP) demonstrating significant clinical relevance in HF populations. Several studies have established its utility in predicting mortality in HF patients and the general population, highlighting its correlation with invasive right heart catheterization measurements and demonstrating its superiority over echocardiographic methods.^3,4^ Moreover, CMR-PCWP has been linked to established markers of myocardial strain, including N-terminal pro B-type natriuretic peptide and major cardiovascular risk factors, including hypertension, male sex, obesity, and aging and has been found to predict HF and major adverse cardiovascular events, suggesting that it should be integrated into routine clinical assessments.^5,6^

Despite advancements, it is unclear if CMR-PCWP, reflecting LVFP, adds predictive value for AF risk beyond established imaging markers.^7^ We hypothesize that elevated CMR-PCWP is associated with an increased incidence of AF and that its predictive value surpasses that of left atrial (LA) volume (LAV) assessment alone.

The primary objective of this study is to evaluate the predictive capacity of CMR-PCWP for incident AF in a high-stroke-risk population. Secondary objectives include comparing the prognostic performance of CMR-PCWP against LAV measures and examining its association with established clinical risk scores for AF. By integrating non-invasive LVFP assessment with continuous rhythm monitoring, this study aims to refine risk stratification for AF and inform targeted preventive strategies.

## Methods

### Study population

Participants for this analysis were drawn from the LOOP study (ClinicalTrials.gov identifier: NCT02036450), a large, randomized trial aimed at detecting and characterizing AF in older adults at heightened stroke risk. To qualify for the main trial, individuals had to be at least 70 years of age, present with one or more recognized stroke risk factors (hypertension, stroke, diabetes mellitus, and HF) and have no history of clinically diagnosed AF. Detailed study design and the documentation of previous findings have been already published.^8,9^ Within this framework, the participants included and randomized to receive implantable loop recorders (ILRs) were offered inclusion in the CMR sub-study as well. A single CMR session was conducted for each enrolled participant after their inclusion in the LOOP study [median time: 25 (14–36) days].

Exclusion criteria for the CMR sub-study specifically encompassed any condition or device that precluded safe and accurate magnetic resonance imaging. This included individuals with ferromagnetic implants, severe claustrophobia, or non-magnetic resonance imaging-conditional devices. With these criteria in place, the investigation provided a focused evaluation of cardiac structure and function in a population at elevated risk for AF but without a prior clinical diagnosis of the arrhythmia.

### Ethics and consent

Every participant provided written informed consent, and the trial adhered to the second Declaration of Helsinki. The protocol was reviewed and approved by the Ethics Committee for the Capital Region of Denmark (protocol number H-4-2013-025) and the Danish Data Protection Agency (no.: 2007-58-0015), with independent oversight throughout.

### Baseline assessment and implantable loop recorder

All patients underwent thorough baseline evaluations, including detailed medical histories, physical examinations, standard electrocardiographic studies, and targeted laboratory testing. Participants also completed standardized assessments of functional status and risk scoring for AF and stroke. Implantable loop recorders (Reveal LINQ™, Medtronic) were implanted in all eligible individuals after a median period time of 23 (15–34) days, enabling continuous cardiac rhythm recording for a median follow-up period of 39 (36–42) months.

### Cardiovascular magnetic resonance protocol

The CMR protocol relied on electrocardiographic gating and was performed on clinical 1.5T scanners (Espree, Siemens Healthcare, Germany). After initial localizer acquisitions, balanced steady-state free precession cine images were obtained to assess ventricular and atrial volumes. Short-axis cine slices (∼8 mm thick; no gap; 25 phases) were acquired from the atrioventricular ring to the apex, measuring LV and right ventricular (RV) volumes, ejection fractions, and LV mass (LVM); papillary muscles were excluded from LVM calculations. Additional cine images were acquired in two-chamber and four-chamber planes to capture maximum and minimum LAVs; the LA appendage and the pulmonary veins were excluded when clearly visible.

### Image analysis

Cardiovascular magnetic resonance image analysis was conducted using CVI^42^ software (Version 5.17.1, Circle Cardiovascular Imaging, Calgary, Canada). Left ventricular parameters—including LV end-diastolic volume (LVEDV, mL), LV end-systolic volume (LVESV, mL), LVM (g) at diastole were quantified using semi-automated contouring techniques applied to short-axis cine images. Manual refinements were performed where necessary to enhance measurement accuracy. Left ventricular volumes and LV ejection fraction (LVEF, %) were determined via Simpson’s method of discs. Left ventricular stroke volume (LVSV, mL) was calculated by the subtraction of LVESV from LVEDV. Left ventricular mass was calculated by subtracting endocardial volume from epicardial volume and multiplying by myocardial tissue density. Right ventricular parameters were assessed similarly. Left atrial volumes (ml) were assessed in end-diastole (LAV min) and end-systole (LAV max) according to the biplane area–length method in both four-chambers and two-chambers axial planes, ensuring the exclusion of pulmonary veins and the atrial appendage. When LA was not visualized in one of these views, the other was used with the same method in one plane. Left atrial total emptying fraction (LAEF, %) was defined as (LAV max-LAV min)/LAV max. All measurements were indexed to body surface area, calculated by DuBois’ formula. All measurements were performed by three operators (M.K., B.C., and P.T.) and reviewed by an experienced CMR consultant (P.P.S.). All operators were blinded with respect to clinical data, including the AF incidence.

### Estimation of cardiovascular magnetic resonance-pulmonary capillary wedge pressure

Pulmonary capillary wedge pressure was estimated using sex-specific equations derived from CMR parameters, as previously established.^10^ This model integrates LAV and LVM, serving as an indirect measure of LVFP. These parameters were incorporated into the sex-specific equation, offering a reliable method for diastolic function evaluation:


$$\begin{array}{rl} \mathrm{CMR}\text{-}\mathrm{PCWP} & =5.7591+(0.07505\times \mathrm{LAV})+(0.05289\times \mathrm{LVM})\\ & \;-(1.9927\times \mathrm{sex})[\mathrm{female}=0;\mathrm{male}=1]. \end{array}$$

where PCWP represents pulmonary capillary wedge pressure (mmHg), LAV is left atrial volume in end systole (ml) when LAV is maximum, LVM denotes left ventricular mass (g), and sex is coded as female = 0, male = 1. This equation provides a sex-specific approach for estimating LVFP, facilitating the assessment of diastolic function using non-invasive CMR-derived metrics.

### Endpoints

During follow-up, heart rhythm data were remotely transmitted to an online database on a day-to-day basis. The monitoring continued until the end of service of the device, patient withdrawal, or death. Electrocardiogram documentation of any arrhythmias was independently evaluated by two different experienced heart rhythm specialists. In case of disagreement, a third expert was involved to provide a majority vote to resolve the event. New-onset AF lasting ≥6 min was defined as AF incidence, which was also the cut-off value of clinical relevant AF in pacemaker patients in the ASSERT trial.^11^ In the current study, our endpoint was the time to AF incidence. In case of AF incidence, antithrombotic treatment with oral anticoagulation (OAC) was initiated promptly based on the decision of the treating physician.

### Statistical analysis

All statistical analyses were conducted using parametric approaches because the sample size was sufficiently large to invoke the central limit theorem. Given our adequate sample and prespecified plan, we used parametric methods and Cox proportional-hazards models, relying on the central limit theorem. Continuous variables were expressed as mean ± standard deviation (SD) and compared between groups using unpaired two-tailed Student’s *t*-tests; categorical variables were summarized as counts and percentages and compared via *χ*^2^ tests. Time-to-event outcomes were assessed using the Kaplan–Meier method, with differences between strata evaluated by the log-rank test; Cox proportional hazards regression models (enter and stepwise methods) were employed to identify independent predictors of AF. Receiver operating characteristic (ROC) curve analysis provided discrimination metrics, with the area under the curve reported. A two-sided *P*-value <.05 was considered statistically significant.

### Sample size calculations

A sample size calculation was performed to assess the adequacy of the study population for detecting between-group differences in CMR-PCWP of ∼2 mmHg (assuming a SD of 4 mmHg, *α* = 0.05, and 80% power). The number of 202 individuals enrolled in the study exceeded the threshold for statistical power, supporting that the sample size was sufficient to detect the expected differences.

## Results

A total of 205 participants randomized to ILR group in LOOP study were included in the CMR sub-study. Participants were excluded if they had missing rhythm monitoring details (*n* = 2) or uninterpretable CMR analysis (*n* = 1), resulting in a study population comprised of 202 patients. In 27 participants, LA was not visualized adequately in four- and two-chambers, and the LA indices were derived from monoplanar measurements.

Over a median follow-up period of 39 (36–42) months with the ILR, 57.4% (*n* = 116) had no AF, and 42.6% (*n* = 86) had developed evidence of AF (*Table 1*). The mean age (75.75 ± 3.85 vs 76.68 ± 4.65, *P* = .12) and the male proportion (66.3% vs 61.2%, *P* = .46) were comparable between groups. No significant differences were observed in anthropometrics, while cardiovascular risk factors were distributed similarly between the two groups. Symptom burden and AF risk scores were comparable, except for the CHARGE-AF score, which was slightly higher in the incident AF group (14.31 ± 0.60 vs 14.11 ± 0.63, *P* = .02). Baseline haemodynamic parameters, including systolic blood pressure, diastolic blood pressure, and heart rate did not differ significantly between the groups.

**Table 1:** Patient demographics

	No AF (*n* = 116)	Incident of AF (*n* = 86)	*P*-values
Patient characteristics			
Age (years)	75.75 ± 3.85	76.68 ± 4.65	.12
Sex (male, %)	71 (61.2%)	57 (66.3%)	.46
Height (cm)	171.34 ± 8.74	173.22 ± 9.18	.14
Weight (kg)	81.76 ± 13.83	85.92 ± 17.44	.06
Body mass index (kg/m^2^)	27.86 ± 4.37	28.58 ± 5.23	.27
Common cardiovascular risk factors			
Smoking (%, yes)	7 (6.0%)	10 (11.6%)	.31
Heart failure (%, yes)	3 (2.6%)	4 (4.7%)	.43
Acute myocardial infarction (%, yes)	10 (8.6%)	8 (9.3%)	.87
Hypertension (%, yes)	104 (89.7%)	79 (91.9%)	.59
Diabetes mellitus (%, yes)	40 (34.5%)	24 (27.9%)	.32
Coronary artery bypass grafting (%, yes)	5 (4.3%)	3 (3.5%)	.77
Syncope (%, yes)	16 (13.8%)	15 (17.4%)	.48
Valvular disease (%, yes)	3 (2.6%)	5 (5.8%)	.25
Chronic obstructive pulmonary disease (%, yes)	9 (7.8%)	3 (3.5%)	.21
Transient ischaemic attack (%, yes)	10 (8.6%)	13 (15.1%)	.15
Stroke (%, yes)	17 (14.7%)	21 (24.4%)	.08
Embolism (%, yes)	8 (6.9%)	5 (5.8%)	.76
Symptom burden and other relevant AF risk scores			
New York Heart Association class (score)	1.16 ± 0.36	1.27 ± 0.52	.07
CHA_2_DS_2_-VASc score (score)	3.8 ± 1.3	4 ± 1.2	.27
CHARGE-AF score (score)	14.1 ± 0.6	14.3 ± 0.6	.02
Baseline haemodynamic assessment			
Systolic blood pressure (mmHg)	148 ± 18	148 ± 17	.88
Diastolic blood pressure (mmHg)	85 ± 12	85 ± 10	.43
Heart rate (beats per minute)	72 ± 12	69 ± 11	.12

In the non-indexed CMR assessment, individuals with incident AF had higher LVEDV (152.9 ± 38.8 vs 138.4 ± 32.0, *P* < .01), LVESV (63.1 ± 23.4 vs 56.4 ± 22.0, *P* = .04), LV stroke volume (LVSV) (89.8 ± 23.2 vs 82.0 ± 16.1, *P* < .01), and LVM (110.6 ± 30.6 vs 100.2 ± 23.6, *P* < .01), while LVEF remained similar (*P* = .52). Right ventricular parameters, including RV end-diastolic volume (RVEDV), RV end-systolic volume (RVESV), and RV stroke volume (RVSV) were also higher in the incident AF group, with no significant difference in RV ejection fraction (RVEF). Left atrial volume max (76.9 ± 25.7 vs 65.3 ± 23.0, *P* < .01) and LAV min (35.9 ± 17.5 vs 27.4 ± 13.6, *P* < .01) were greater in the incident AF group, accompanied by a lower LAEF (55.0 ± 9.3 vs 58.9 ± 9.6, *P* < .01). Indexed CMR assessment revealed similar results. Cardiovascular magnetic resonance-pulmonary capillary wedge pressure was higher in the incident AF group (16.1 ± 2.8 vs 14.7 ± 2.3, *P* < .01). Receiver operating characteristic curve analysis was employed to determine the optimal CMR-PCWP threshold in predicting AF, identifying 16 mmHg as the cut-off value. Patients who developed AF showed a significantly higher prevalence of CMR-PCWP ≥16 mmHg compared with those without AF (46.5% vs 19.8%, *P* < .01) (*Table 2*).

**Table 2:** Comparison of cardiac magnetic resonance parameters between individuals without atrial fibrillation and those with incident atrial fibrillation

CMR parameters	No AF (*n* = 116)	Incident AF (*n* = 86)	*P*-value
Non-indexed CMR assessment			
LVEDV (ml)	138.4 ± 32.0	152.9 ± 38.8	<.01
LVESV (ml)	56.4 ± 22.0	63.1 ± 23.4	.04
LVSV (ml)	82.0 ± 16.1	89.8 ± 23.2	<.01
LVM (g)	100.2 ± 23.6	110.6 ± 30.6	<.01
LVEF (%)	60.0 ± 7.2	59.3 ± 8.1	.52
RVEDV (ml)	136.0 ± 30.3	150.7 ± 35.6	<.01
RVESV (ml)	57.9 ± 18.0	64.8 ± 20.6	.01
RVSV (ml)	78.1 ± 17.1	85.9 ± 20.5	<.01
RVEF (%)	57.9 ± 7.0	57.5 ± 6.7	.66
LAV max (ml)	65.3 ± 23.0	76.9 ± 25.7	<.01
LAV min (ml)	27.4 ± 13.6	35.9 ± 17.5	<.01
LAEF (%)	58.9 ± 9.6	55.0 ± 9.3	<.01
CMR- PCWP (mmHg)	14.7 ± 2.3	16.1 ± 2.8	<.01
Indexed CMR assessment			
LVEDV*_i_* (ml/m^2^)	71.2 ± 13.9	76.5 ± 16.6	.01
LVESV*_i_* (ml/m^2^)	28.8 ± 9.7	31.5 ± 11.0	.07
LVSV*_i_* (ml/m^2^)	42.4 ± 8.0	45.0 ± 10.0	.04
LVM*_i_* (g/m^2^)	51.3 ± 9.5	55.0 ± 11.2	.01
RVEDV*_i_* (ml/m^2^)	69.3 ± 14.7	75.4 ± 14.9	<.01
RVESV*_i_* (ml/m^2^)	29.3 ± 8.4	32.2 ± 8.7	.02
RVSV*_i_* (ml/m^2^)	40.3 ± 8.2	43.3 ± 9.3	.02
LAV max*_i_* (ml/m^2^)	33.6 ± 11.2	38.5 ± 11.6	<.01
LAV min*_i_* (ml/m^2^)	14.1 ± 6.6	17.9 ± 7.9	<.01

LVEDV, left ventricular end-diastolic volume; LVESV, left ventricular end-systolic volume; LVSV, left ventricular stroke volume; LVM, left ventricular mass; LVEF, left ventricular ejection fraction; RVEDV, right ventricular end-diastolic volume; RVESV, right ventricular end-systolic volume; RVEF, right ventricular ejection fraction, LAV max, left atrial volume maximum; LAV min, left atrial volume minimum; LAEF, left atrial total emptying fraction; LVEDV*_i_*, left ventricular end-diastolic volume index; LVESV*_i_*, left ventricular end-systolic volume index, LVSV*_i_*, left ventricular stroke volume index; LVM*_i_*, left ventricular mass index; RVEDV*_i_*, right ventricular end-diastolic volume index; RVESV*_i_*, right ventricular end-systolic volume index; RVSV*_i_*, right ventricular stroke volume index; LAV max*_i_*, left atrial volume maximum index; LAV min*_i_*, left atrial volume minimum index; CMR-PCWP, cardiac magnetic resonance-derived pulmonary capillary wedge pressure.

### Univariate Cox-regression survival analysis

**Figure 1:**
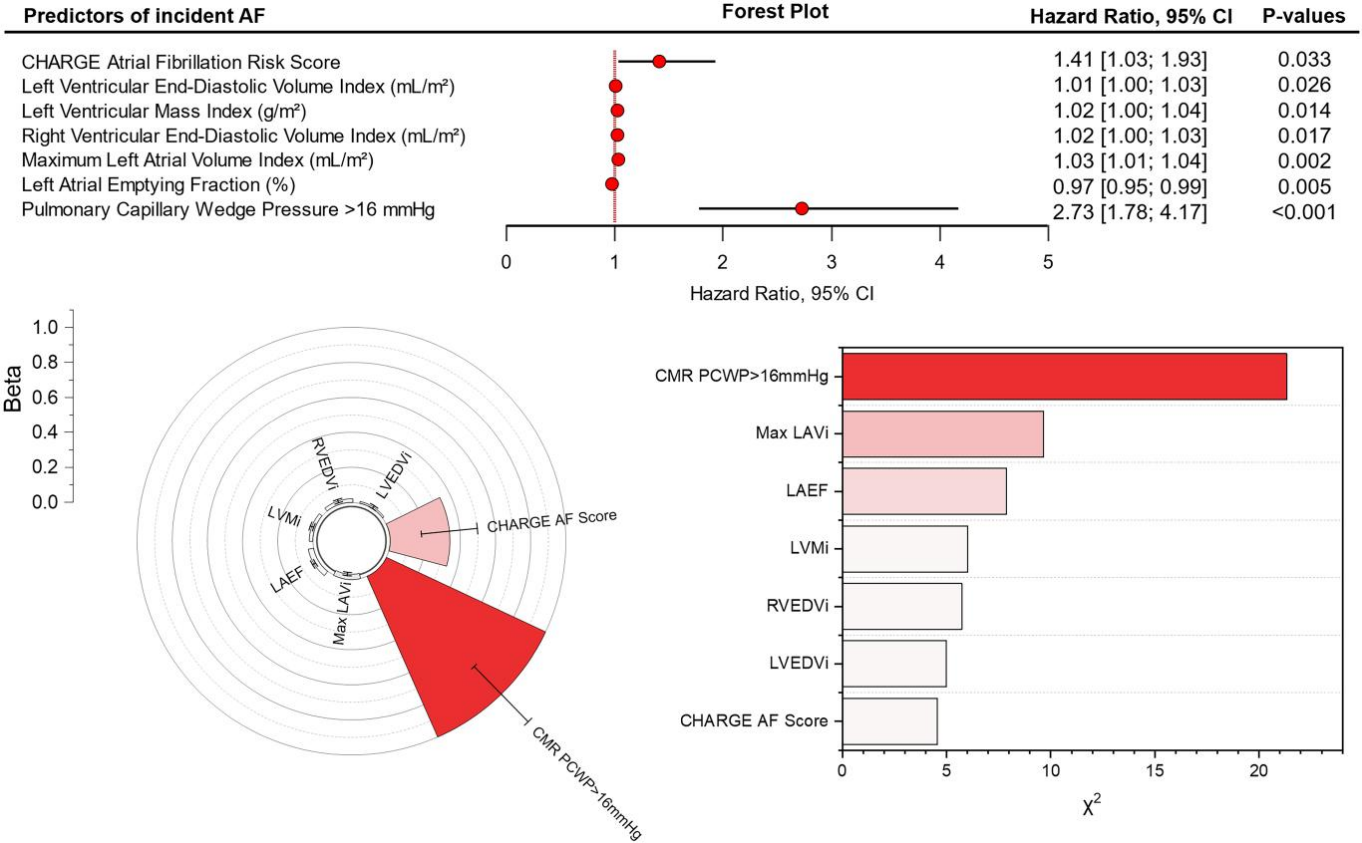
Univariate time-to-event analysis identifying predictors of incident atrial fibrillation. The forest plot (top) displays hazard ratios with 95% confidence intervals for various cardiac parameters, demonstrating that pulmonary capillary wedge pressure (>16 mmHg) is the strongest predictor (hazard ratio: 2.73, *P* < .001). The radial plot (bottom left) illustrates the relative effect size (beta coefficients) of predictors, highlighting the dominant contribution of pulmonary capillary wedge pressure. The bar chart (bottom right) ranks predictors based on their chi-square (*χ*^2^) values, confirming pulmonary capillary wedge pressure as the most significant variable for atrial fibrillation risk stratification. AF, atrial fibrillation; CI, confidence interval; HR, hazard ratio; CMR-PCWP, cardiac magnetic resonance-derived pulmonary capillary wedge pressure; LAV max*_i_*, left atrial volume index; LAEF, left atrial emptying fraction; LVM*_i_*, left ventricular mass index; RVEDV*_i_*, right ventricular end-diastolic volume index; LVEDV*_i_*, left ventricular end-diastolic volume index

The CHARGE-AF score demonstrated a significant association with AF occurrence in this high-risk patient population [*β* = 0.34, hazard ratio (HR): 1.41, 95% confidence interval (CI): 1.03–1.93, *P* = .03] (*Figure 1*). For CMR, increased LVEDV*_i_* (*β* = 0.01, HR: 1.01, 95% CI: 1.00–1.03, *P* = .03), LVM*_i_* (*β* = 0.02, HR: 1.02, 95% CI: 1.00–1.04, *P* = .01), and RVEDV*_i_* (*β* = 0.02, HR: 1.02, 95% CI: 1.00–1.03, *P* = .02) were significantly associated with AF. Left atrial volume max*_i_* exhibited a positive association with AF (*β* = 0.03, HR: 1.03, 95% CI: 1.01–1.04, *P* = .002), while LAEF was inversely associated with AF risk (*β* = −0.03, HR: 0.97, 95% CI: 0.95–0.99, *P* = .004). The highest HR was seen for CMR-PCWP ≥ 16 mmHg (*β* = 1.00, HR: 2.73, 95% CI: 1.78–4.17, *P* < .001; *Table 3*).

**Table 3:** Univariate Cox-regression time to event analysis

Cardiac magnetic resonance variables	Coefficient (beta)	Standard error	*P*-value
CHARGE-AF score	0.34	0.16	.033
LVEDV*_i_* (ml/m^2^)	0.01	0.01	.026
LVM*_i_* (g/m^2^)	0.02	0.01	.014
RVEDV*_i_* (ml/m^2^)	0.02	0.01	.017
LAV max*_i_* (ml/m^2^)	0.03	0.01	.002
LAEF (%)	−0.03	0.01	.005
CMR-PCWP ≥16 mmHg	1	0.22	<.001

CHARGE, Cohorts for Heart and Aging Research in Genomic Epidemiology; AF, atrial fibrillation; LVEDV*_i_*, left ventricular end-diastolic volume index; LVM*_i_*, left ventricular mass index; RVEDV*_i_*, right ventricular end-diastolic volume index; LAV max*_i_*, left atrial volume maximum index; LAEF, left atrial emptying fraction; CMR-PCWP, cardiac magnetic resonance derived-pulmonary capillary wedge pressure.

### Receiver operating characteristic analysis

**Figure 2:**
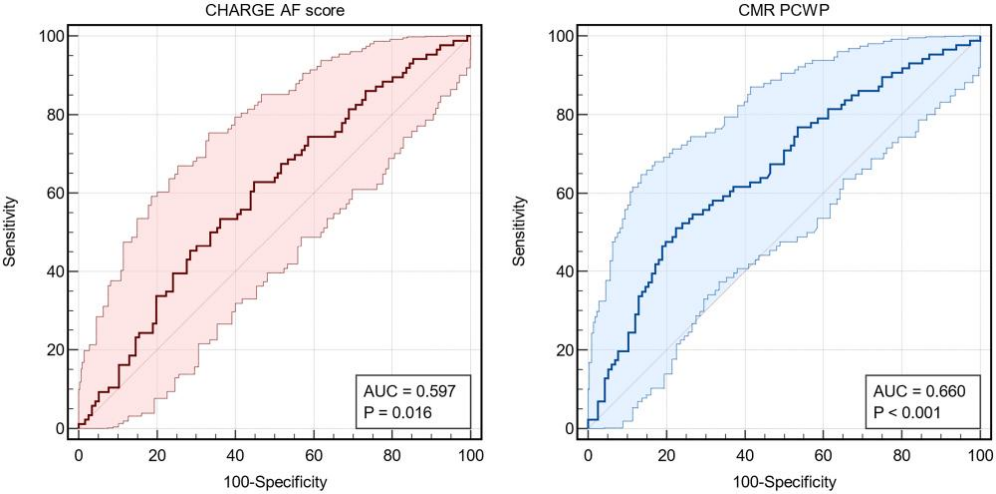
Receiver operating characteristic curves for predicting future atrial fibrillation using the CHARGE-AF score and cardiovascular magnetic resonance-pulmonary capillary wedge pressure. Shaded areas indicate 95% confidence intervals. Area under the curve values and corresponding *P*-values are displayed for each model. CHARGE-AF score, Cohorts for Heart and Aging Research in Genomic Epidemiology—Atrial Fibrillation score; CMR-PCWP, cardiovascular magnetic resonance-derived pulmonary capillary wedge pressure

Predictive performance for future AF on ILR was assessed using ROC curve analysis. The CHARGE-AF score demonstrated an area under the curve (AUC) of 0.597 (*P* = .016), while CMR-PCWP yielded a higher AUC of 0.660 (*P* < .001) (*Figure 2*). The CIs indicate greater variability in CHARGE-AF score predictions, whereas CMR-PCWP exhibited a more robust discriminative ability.

### Kaplan–Meier survival analysis

**Figure 3:**
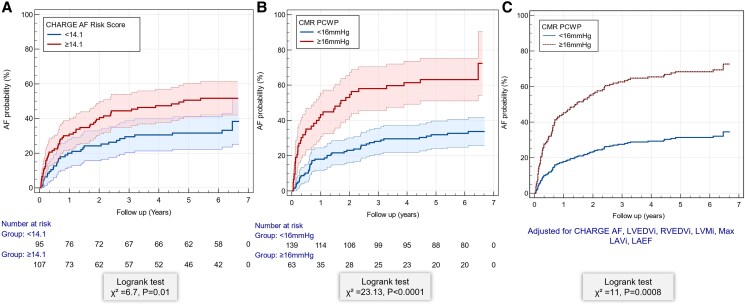
Kaplan–Meier curves for atrial fibrillation probability stratified by (A) CHARGE-AF score and (B) Cardiovascular magnetic resonance-pulmonary capillary wedge pressure. (C) Cox regression for pulmonary capillary wedge pressure, adjusted for CHARGE-AF score, left ventricular end-diastolic volume index, right ventricular end-diastolic volume index, left ventricular mass index, left atrial volume maximum Index, and left atrial emptying fraction. Shaded areas indicate 95% confidence intervals. Log-rank test results are shown. AF, atrial fibrillation; KM, Kaplan–Meier; CMR-PCWP, cardiovascular magnetic resonance-derived pulmonary capillary wedge pressure; LVEDV*_i_*, left ventricular end-diastolic volume index; RVEDV*_i_*, right ventricular end-diastolic volume index; LVM*_i_*, left ventricular mass index; LAVmax*_i_*, left atrial volume maximum index; LAEF, left atrial emptying fraction

Kaplan–Meier analysis demonstrated a significantly higher probability of AF in individuals with CHARGE-AF scores ≥14.1 compared with those with scores <14.1 (*χ*^2^ = 6.7, *P* = .01; *Figure 3*). Similarly, patients with CMR-PCWP ≥16 mmHg exhibited a significantly greater AF probability over time than those with PCWP <16 mmHg (*χ*^2^ = 23.13, *P* < .0001). Cox proportional hazards regression adjusted for CHARGE-AF score, LVEDV*_i_*, RVEDV*_i_*, LVM*_i_*, LAV max*_i_*, and LAEF, confirmed that elevated PCWP remained a significant predictor of AF (*χ*^2^ = 11, *P* = .0008).

### Important predictors of future incidence of atrial fibrillation

The multivariate Cox regression stepwise time-to-event analysis, incorporating the CHARGE-AF score and multiple CMR parameters, identified two independent covariates associated with future AF incidence on ILR recording. Cardiovascular magnetic resonance-pulmonary capillary wedge pressure ≥16 mmHg was associated with a HR of 2.88 (95% CI: 1.87–4.43, *P* < .001), while the CHARGE-AF score demonstrated a HR of 1.48 (95% CI: 1.06–2.09, *P* = .023). Several variables, including LAEF, LAV max_i_, LVED*_i_*, and RVEDV*_i_*, were removed during stepwise regression. The overall model fit was significant (*χ*^2^ = 26.472, df = 2, *P* < .0001; *Table 4*). Overall, 63 patients had CMR-PCWP ≥16 mmHg, and 63.5% (40/63) of those had AF, compared with only 33% of patients with CMR-PCWP <16 mmHg (*P* = .0001).

**Table 4:** Multivariate Cox-regression stepwise time-to-event analysis factoring CHARGE-AF score and several cardiac magnetic resonance parameters

Covariate	Beta	SE	Wald	Exp (*β*)	95% CI low	95% CI high	*P*-value
CMR-PCWP ≥16 mmHg	1.06	0.22	23.04	2.88	1.87	4.43	<.001
CHARGE-AF score	0.4	0.17	5.17	1.48	1.06	2.09	.023
Variables removed in stepwise Cox regression: Left atrial emptying fraction Left atrial volume index Left ventricular end-diastolic volume index Left ventricular mass index Right ventricular end-diastolic volume index							
Overall model fit	*χ* ^2^ = 26.472 (df = 2), *P* < .0001						

CMR-PCWP, cardiovascular magnetic resonance-derived pulmonary capillary wedge pressure; CHARGE, Cohorts for Heart and Aging Research in Genomic Epidemiology; CI, confidence interval; SE, standard error; Exp (β), exponentiated beta (hazard ratio); df, degrees of freedom; *χ*^2^, chi-square.

Using CMR-PCWP as a continuous variable, it was also demonstrated to remain independently associated with future AF risk (HR: 1.3, 95% CI: 1.01–1.6, *P* = .04). The only other parameter which remained independently associated was the CHARGE-AF score—again demonstrating how CMR-PCWP provides incremental value over routine CMR and other clinical characteristics.

## Discussion

**Figure 4:**
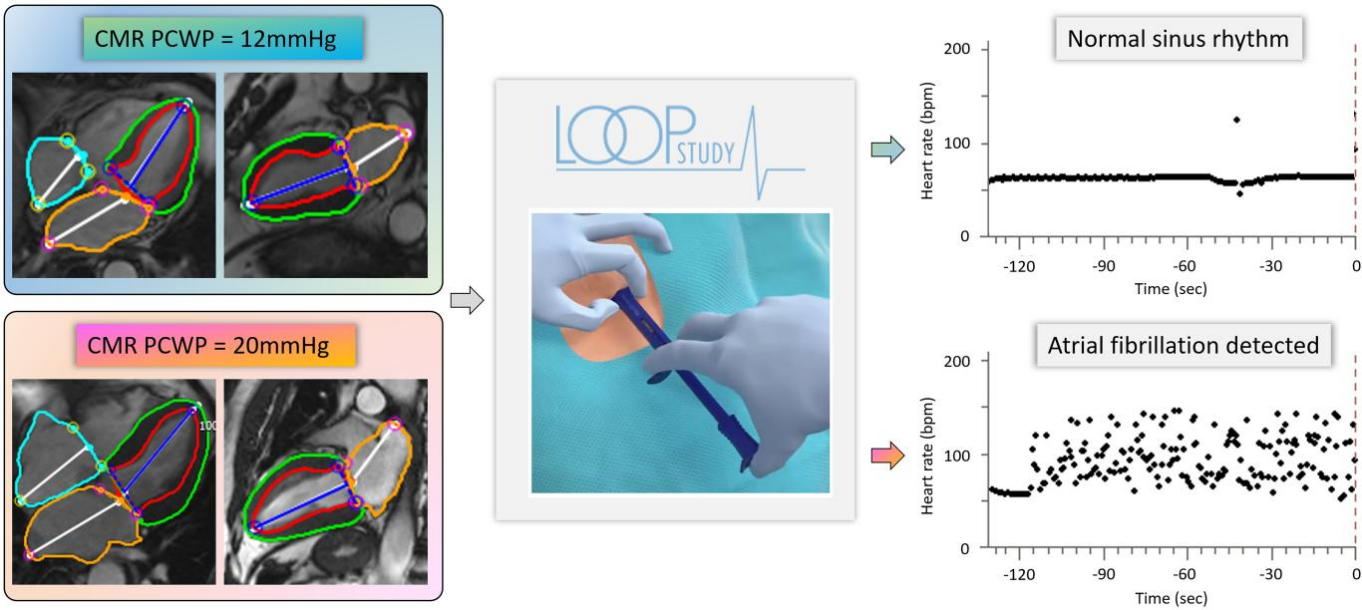
Cardiovascular magnetic resonance image analysis of two separate patients with normal and increased cardiovascular magnetic resonance-pulmonary capillary wedge pressure, respectively, and their implantable loop recorder rhythm monitoring data, indicating the incidence of atrial fibrillation in the case of increased cardiovascular magnetic resonance-pulmonary capillary wedge pressure. CMR-PCWP, cardiovascular magnetic resonance-derived pulmonary capillary wedge pressure; ILR, implantable loop recorder; AF, atrial fibrillation

The main findings of this study are that in older adults at elevated stroke risk yet free from a prior AF diagnosis, a non-invasive CMR-PCWP is a strong indicator of future AF (*Figure 4*). Individuals with CMR-PCWP ≥16 mmHg faced a nearly threefold greater hazard of developing subclinical AF compared with those below this threshold, reflecting that even mild elevations in LVFP can substantially elevate arrhythmic risk (*Graphical Abstract*). More nuanced analyses revealed that LA enlargement and diminished emptying fraction, although variably contributory, had less prognostic weight than CMR-PCWP index. These observations suggest that subclinical diastolic dysfunction exerts a pivotal influence on AF onset in this population and highlight the practical utility of CMR-based haemodynamic assessment for targeted early intervention.

### Non-invasive prediction of atrial fibrillation outcomes

In comparison to our study, Kawasaki *et al*.^12^ used speckle tracking echocardiography (STE) to estimate PCWP in 137 patients with paroxysmal AF, linking it to successful ablation with an odds ratio of 0.482 per mmHg increase (*P* < .0001). Similar to our analysis, this study did not show a predictive advantage of ablation success with individual LA parameters, highlighting that composite PCWP is a better imaging marker to predict atrial arrhythmogenicity. In another study, called the NIPAF study by the same authors, they followed 566 participants over 4 years, associating speckle tracking echocardiography-derived PCWP with new-onset non-valvular AF.^12^ Their AF pick up rates, however, were much lower than our study at 11% only, even though the study cohorts were similar. Another important difference between their and our study is they used clinical criteria for AF vs investigating subclinical AF. They used standard 12-lead electrocardiograms or Holter if patient became symptomatic. Therefore, this discrepancy was very likely due to a lack of continuous AF monitoring in their study vs this study, in which patients were given prospective ILRs to record any evidence of AF.

Previous multi-ethnic study of atherosclerosis (MESA) sub-studies have leveraged CMR imaging to predict incident AF, demonstrating that CMR-derived measures independently enhance risk stratification beyond traditional factors.^13,14^ In adjusted Cox regression models, an average annualized change in all LA parameters was significantly associated with an increased risk of AF. However, their study differs significantly in approach and context: our analysis focuses on a single, threshold-based predictor—CMR-PCWP ≥16 mmHg—in older adults at high stroke risk, using continuous ILR monitoring for sensitive AF detection. In contrast, the MESA study assesses longitudinal changes in multiple LA parameters in a younger, multi-ethnic cohort initially free of cardiovascular disease, relying on less sensitive administrative data for AF identification. Because of this, the incidence of AF in their population was only 5% over a follow-up period of 3.8 years. While our study offers a practical, targeted tool for high-risk populations, the MESA study provides a broader, dynamic perspective on LA remodelling. However, its complexity may limit immediate clinical application.

In contrast to our study, Gunasekaran *et al*.^15^ determined that LV extracellular volume (ECV) is a quantitative indicator of myocardial fibrosis and investigated if it is associated with AF recurrence post catheter ablation. In their observations, mean ECV values were comparable between patients maintaining sinus rhythm and those experiencing AF recurrence (25.1% vs 24.7%, respectively), suggesting that ventricular fibrosis may not substantially contribute to the persistence of AF post-intervention. This divergence in predictive capacity likely reflects the distinct pathophysiological roles of these parameters: PCWP directly correlates with LA haemodynamic stress, a primary driver of atrial structural and electrical changes implicated in AF initiation, whereas LV ECV predominantly signifies ventricular fibrosis, which appears less relevant to atrial electrophysiology or the maintenance of AF following ablation.^16^ Additionally, although LAV emerged as a significant correlate of incident AF in our cohort, it did not differentiate between patients with and without AF recurrence in the post-ablation population studied by Gunasekaran *et al*. This observation suggests that the mechanisms governing the onset of AF—potentially more dependent on preload and atrial stretch—differ from those influencing its recurrence, which may be more closely tied to ablation-specific outcomes and the pre-existing irreversible atrial substrate.

### Atrial fibrillation substrate: role of afterload assessment

Other studies have explored imaging markers beyond PCWP to predict AF, aligning with the present study’s preventive focus but differing methodology. Chrispin *et al*.^17^ analysed 4942 participants in MESA, finding that LV hypertrophy (LVH) on CMR predicted incident AF with an adjusted HR of 2.04 (95% CI: 1.15–3.62). This study furthers the notion that AF substrate is linked not only with pre-loading conditions on the ventricle, which is usually assessed by LAV and function but also with the afterload conditions on the LV, which eventually leads to LVH.

### Limitations

This study’s cohort consisted primarily of older subjects with elevated stroke risk, raising questions about applicability to broader populations. Yet, such individuals constitute the demographic with the highest AF incidence, reinforcing the clinical pertinence of these findings. The CMR-PCWP estimation hinges on a sex-specific formula involving LAV and LVM, potentially subject to measurement variance relative to invasive standards; however, prior validations and the robust predictive accuracy exhibited here underscore its translational value.

At present, there is no universally accepted duration^18^ of device-detected subclinical AF that should trigger OAC use due to heterogeneity in clinical trial outcomes.^19,20^ This is likely due to variation in study comorbidities resulting in differing CHA_2_DS_2_-VASc scores, as well as differing detection algorithms and sensitivities. Though ILRs provide the gold standard in continuous rhythm monitoring, very short durations of AF and R-wave undersensing can reduce sensitivity. The present study utilized a ≥6 min detection threshold from the original LOOP study,^9^ which was concordant with the findings of the ASSERT trial where AF duration of ≥6 min was clinically significant.^11^ In a less comorbid population, longer durations of AF might be required for significance, which could affect the threshold for CMR-PCWP.

## Conclusions

Elevated CMR-PCWP is strongly associated with AF incidence in older, high-stroke-risk individuals by reflecting subclinical diastolic dysfunction. Incorporating CMR-PCWP into routine risk stratification could refine early detection and guide targeted interventions for AF prevention in this vulnerable population.

## Declarations

### Data Availability

The article's data will be shared on reasonable request to the corresponding author.

### Pre-registered Clinical Trial Number

None supplied.
